# Reply to “Correspondence regarding ‘Obstructive sleep apnea and primary snoring in children are associated with oropharyngeal dysbiosis and a mild compositional imbalance in the gastrointestinal tract’”

**DOI:** 10.1007/s44470-026-00141-4

**Published:** 2026-07-20

**Authors:** Jennifer Hudson, Azleena Akhand, Moe Thant Nwe, Michael J. Coffey, Josie van Dorst, Sandra Chuang, Chee Y. Ooi

**Affiliations:** 1 School of Clinical Medicine, Discipline of Pediatrics and Child Health, The University of New South Wales, Sydney, Australia; 2 Department of Gastroenterology, Sydney Children’s Hospital, Randwick, Australia; 3 Department of Respiratory Medicine, Sydney Children’s Hospital, Randwick, Australia

We thank Dr. Hyun Jin Min for the interest in our article [1] and for the thoughtful comments provided [2]. Dr. Min’s comments highlight several important considerations regarding the interpretation of the observed microbiome associations, and we welcome the opportunity to provide clarification.

We agree that oropharyngeal microbial communities can be influenced by numerous factors, including recent food intake, oral hygiene practices, and the timing of sample collection which may contribute to variability in sequencing-based microbiome analyses [3]. To clarify our methodology, all oropharyngeal swabs were collected during daytime clinic hours, prior to the commencement of the overnight sleep study, at approximately 4 pm. Information regarding recent dietary intake and oral hygiene practices was not collected due to practical challenges of standardizing and enforcing strict oral protocols in a young pediatric cohort. We acknowledge this as a limitation of the study, as some taxa identified within the oropharyngeal microbiome may reflect transient environmental exposures rather than stable colonization. Further studies employing longitudinal sampling will be needed to characterize the stable microbial community in children with sleep-disordered breathing (SDB).

We also agree that allergic and upper airway inflammatory conditions may influence the composition of oral microbial communities in children with SDB [4, 5]. Information regarding allergic and upper airway inflammatory conditions was not systematically collected in our cohort prospectively and therefore not evaluated in the present analysis. Our study sought to determine whether previously reported microbiome alterations in children with obstructive sleep apnea (OSA) occurred across the spectrum of SDB (including primary snorers) and whether these alterations were associated with measures of disease severity such as obstructive apnea/hypopnea index or SpO_2_ nadir. Disentangling the relative contributions of SDB and upper airway inflammation on the microbial community structure, as well as identifying the underlying mechanisms of oropharyngeal dysbiosis, will require further investigation.

Our results demonstrated that, within this cohort, the airway microbiomes of children with OSA and primary snoring were broadly similar and distinct from healthy controls. These observations suggest that factors common to both groups may contribute to the observed microbiome alterations, rather than OSA severity or hypoxic burden alone. Whilst the specific underlying mechanisms remain uncertain, our findings raise the possibility that shared features of SDB, including altered breathing patterns, upper airway anatomy, and local inflammatory processes, may influence the microbial community structure. We agree with Dr. Min that adenotonsillar hypertrophy represents a relevant factor, along with other anatomical variables such as tongue position and craniofacial differences between individuals [6, 7]. Although stratifying individuals according to adenotonsillar size may provide an important insight into the link between upper airway obstruction and microbial dysbiosis, reliable and objective assessments of adenotonsillar size were not routinely available. Grading tonsillar size visually using various tonsillar size grading scales remains observer-dependent with variation across raters [8]. Otolaryngology review and radiological studies were not undertaken in all children included in the present study, particularly those classified as primary snorers. Nevertheless, the similarity in microbial profiles observed between children with OSA and primary snoring suggests factors common to both conditions may be important determinants of airway microbial composition. Identifying these factors, including upper airway inflammation, anatomical factors such as adenotonsillar hypertrophy, and altered breathing patterns, represents an important next step in determining the mechanisms underpinning airway dysbiosis in children with sleep-disordered breathing.

We thank Dr. Hyun Jin Min again for these valuable comments and for highlighting important directions for future research.

## Data Availability

Sequencing data associated with the original publication is deposited in the NCBI Sequence Read Archive (SRA) under the BioProject PRJNA1312271. No new data was generated as part of this correspondence.
